# Allergological and Toxicological Aspects in a Multiple Chemical Sensitivity Cohort

**DOI:** 10.1155/2013/356235

**Published:** 2013-12-03

**Authors:** Paolo D. Pigatto, Claudio Minoia, Anna Ronchi, Lucia Brambilla, Silvia M. Ferrucci, Francesco Spadari, Manuela Passoni, Francesco Somalvico, Gian Paolo Bombeccari, Gianpaolo Guzzi

**Affiliations:** ^1^Department of Bioscience for Health, Dermatological Clinic, IRCCS Galeazzi Hospital, University of Milan, 20161 Milan, Italy; ^2^Laboratory of Environmental and Toxicology Testing “S. Maugeri” IRCCS, 27100 Pavia, Italy; ^3^Operative Unit of Dermatology, Fondazione IRCCS Ca' Granda-Ospedale Maggiore Policlinico, 20122 Milano, Italy; ^4^Department of Biomedical, Surgical and Dental Sciences, Unit of Oral Pathology and Medicine, Ospedale Maggiore Policlinico Fondazione Ca' Granda IRCCS, University of Milan, 20122 Milan, Italy; ^5^Italian Association for Metals and Biocompatibility Research (AIRMEB), 20122 Milan, Italy; ^6^Alpha Search, 20063 Milan, Italy

## Abstract

*Background*. Multiple chemical sensitivity (MCS) is a chronic condition characterized by an exaggerated response to toxicants. We ascertained the prevalence of allergy to metals and toxicological aspects in MCS patients. *Methods*. We conducted a retrospective review of medical records of 41 patients with MCS. We performed patch testing (*n* = 21) for dental series and did lymphocyte transformation test (*n* = 18) for metals. We measured mercury in samples of blood (*n* = 19), urine (*n* = 19), saliva (*n* = 20), and scalp hair (*n* = 17) to investigate the association between mercury levels and cases of MCS. *Results*. The prevalence of metal immune hypersensitivity in a subset of 26 patients was 92.3 percent. Elevations of mercury occurred in 81.2 percent (26 of 32). The mean (±SD) in blood concentrations of mercury was 7.6 ± 13.6 **μ**g/L; mean in urine was 1.9 ± 2.5 **μ**g/L; mean in scalp hair was 2.2 ± 2.5 **μ**g/g; mean in saliva was 38.1 ± 52.1 **μ**g/L. Subgroup analyses showed that elevation of mercury levels in biological matrices were associated with mercury amalgams in patients with MCS (22 patients), compared with controls (8 patients) (odds ratio 11 : 95 percent confidence interval 1.5 to 81.6; *P* = 0.023). *Conclusions*. Our data show an increased prevalence of metal allergy and elevation of mercury levels in bioindicators among patients with MCS.

## 1. Introduction

Multiple chemical sensitivity (MCS)—also termed idiopathic environmental intolerance (IEI)—is a chronic condition characterized by an exaggerated body response to chemical toxicants, especially organic solvents [1–6]. The symptoms experienced and reported by patients are usually of the respiratory, musculoskeletal [7], and gastrointestinal tracts [8–12]. Symptoms of hyperosmia [13] of the brain are commonly described in patients with MCS when exposed to chemical substances [14, 15]. Thus, it has long been thought that MCS has a strong environmental component [16–20]. The prevalence of MCS is reported to range from 10 to 15 percent in the general population [21, 22] and its pathogenesis remains elusive. Numerous mechanisms have been implicated in the etiologic process of MCS, including *N*-methyl-D-aspartate (NMDA) sensitization [23, 24], peroxynitrite and nitric oxide elevation [24, 25], oxidative stress [26, 27], proinflammatory cytokines [26, 27], altered redox enzymes [26, 27], cytochrome P450 metabolism [26–28], hypoxia [29], serotonin receptors [30], neural sensitization [17, 31–35], and neurogenic inflammation [36]. As a consequence, various studies have suggested that persistent symptoms of MCS impaired health-related quality of life in these patients [37, 38]. And there is evidence that mercury exposure may cause symptoms that clinically overlap with MCS. To test this hypothesis, we conducted a retrospective cohort study to determine (i) whether patients with MCS had detectable levels of mercury due to chronic environmental metal exposure and (ii) whether patients with MCS had allergy to metals since we hypothesized a priori that these exposures might play a causal role in MCS. Our aim, therefore, was to measure the levels of mercury in human biological matrices. We also sought to determine the outcome of allergic reactions to metals by using *in vivo* patch testing and/or *in vitro* lymphocyte transformation test (LTT).

## 2. Materials and Methods

### 2.1. Study Oversight

We evaluated 41 consecutive patients who had been referred for MCS (39 women and 2 men); mean [±SD] age of the patients at their first study visit was 44.8 ± 11.2 years. To study metal immune hypersensitivity, we did *in vivo* patch testing for dental series in 21 subjects, and we also did *in vitro* lymphocyte transformation test (LTT) for 20 metal allergens in 18 case subjects. We analyzed for concentrations of total mercury (elemental, inorganic, and organic mercury) in samples of peripheral whole blood, urine, saliva, and scalp hair and we tested for an association between alterations in the levels of mercury and MCS by means of inductively coupled plasma mass spectrometry method and/or atomic absorption spectrometry. The total number of silver-mercury amalgam restorations was charted, and we recorded when amalgam restorations were placed in teeth in order to correlate approximately the median duration of dental amalgam exposure (Table 1). None of the patients had been occupationally exposed to mercury.

### 2.2. Definition and Clinical Diagnostic Criteria of MCS

MCS is characterized by adverse health effects related to—or exacerbated by—exposure to chemical substances. The most common chemicals triggers of MCS are organic solvents, metals, volatile organic compounds (VOCs), chlorine, drugs, perfumes, hairsprays, diesel exhaust fumes, and pesticides. MCS was defined by the presence of following MeSH code: D018777. Even though an accurate classification remains difficult, criteria for entry in our cohort were similar to those described by Cullen [1, 2, 39]. Of the 53 patients evaluated for the study, 41 were eligible and we excluded 12 patients (12 of 53, 41) who had reported a probable MCS but they did not meet the above classification criteria [1, 2, 39].

### 2.3. Immunological Assay and Immune Markers

Allergic sensitization to metals contained in mercury dental amalgam restorations was ascertained on the basis of patch testing done on the upper back. Most of the patients (*n* = 21) were patch tested with the Italian dental screening series (SIDAPA) obtained from Chemotechnique Diagnostics AB (Malmöe, Sweden). Readings were taken on day 2 (48 hours) and day 4 (96 hours). With regard to the pathophysiology of allergic reactions induced by metal allergens, they are most frequently mediated by type IV immune reactions (delayed-type hypersensitivity reaction), according to the Gell and Coombs classification. Patch testing was integrated with the lymphocyte transformation test (LTT), a noninvasive test *in vitro* to determine cell-mediated immunological responses to metals and metalloids [40]. In most of the patients (*n* = 18), we used a highly sensitive and optimized LTT method in human lymphocytes (LTT-MELISA^®^ - Memory Lymphocyte Immune Stimulation Assay) [41]. Data were expressed as a stimulation index (SI), which was calculated from the quotient of test counts per minute (cpm) and the average cpm from three negative controls [40, 41]. No corticosteroids and/or immunosuppressive drugs were taken for at least two months before skin contact patch-test allergens and/or the LTT. No subjects had used nonsteroidal antiinflammatory drugs (NSAIDs), antibiotics, and H_1_-anti-histamine agents within the previous three weeks before patch testing and/or the LTT. None of these medications were permitted for all routes of administration (enteral, parenteral, topical, and/or by inhalation), which may alter the immunological response to metals.

### 2.4. Sampling and Mercury Analysis of Biological Specimens

We have measured total mercury concentrations in whole blood samples and urine samples in 19 of 41 patients (46.3%) with MCS (Table 3). At the time of admission, fasting morning venous peripheral whole blood samples (4 milliliters) were collected in mercury-free polypropylene tubes containing potassium EDTA (K_2_-EDTA), as an anticoagulant. First morning urine specimens (100 milliliters) and/or 24-hour urine collection specimens were obtained and stored at +4°C until mercury analysis. All blood and urine samples were delivered immediately to the laboratory of toxicology for mercury analysis and were processed within 24–72 hours after collection. Concentrations of total mercury in whole blood, urine, and chewing gum-stimulated whole saliva samples in our cohort were measured by cold vapor atomic absorption spectrometry (CVAAS) [42, 43] and/or ICP-MS (inductively coupled mass spectrometry) methods [43]. The lower limit of detection (LD) for total mercury in both blood and urine was 0.05 micrograms per liter. The intraassay and interassay coefficients of variation, determined at various concentrations, were 2 percent and 5 percent, respectively. External and internal quality-control procedures were made. We also determined total mercury in head hair in a small subgroup of 17 subjects (17 of 41, 41.4 percent), and human scalp hair samples were taken from the occipital region of the head with sterile stainless steel surgical scissors. Each specimen of head hair was collected from the first three centimeters (3 centimeters hair segments) in length next to the scalp, and it was weighed. The overall mean [±SD] weight of the hair specimens was approximately 220 ± 110 milligrams. Mercury hair analysis is proper indicator medium for determining organic mercury, and it reflects exposure that occurred during the last few months. Total mercury in strands of scalp hair was measured with ICP-MS system. The detection limit (DL) of mercury in scalp hair was 0.07 micrograms per gram [44]. To avoid loss of mercury in head hair, none of the 17 patients received hair dyes three months before the hair sampling procedure. Toenails samples were collected from only one subject to confirm the level of exposure to mercury (data not shown). To examine whether there were significant elevations in total mercury levels in saliva, we collected stimulated saliva specimens after chewing a sugar-free gum for 5–10 minutes before collection, and subsequently mercury content was quantified by atomic absorption spectrometry (AAS) [45]. The operational lower limit of detection (LD) for mercury in saliva was 0.1 micrograms per liter [46]. To assess average fish or seafood consumption, we used a semiquantitative food-frequency questionnaire, which included distinct questions about other dietary variables (i.e., alcohol and—as contribution to the total caffeine intake—coffee, tea, and chocolate). We also estimated modifiable risk factors (i.e., smoking) for MCS among patients, using data derived from interviews, questionnaire [47], and the medical records. All subjects gave oral and written informed consent. This retrospective observational case series study was conducted from 2001 to February 5, 2013, which was the cutoff date for analyses of all cases of MCS. The corresponding author gathered the data and vouch for its accuracy.

### 2.5. Interventions

Our team developed a new method to remove mercury amalgam fillings by using the *en bloc technique *[48]. Treatment method includes complete dental amalgam removal. It is good clinical practice to avoid unnecessary overexposure to mercury vapor during the removal of dental amalgam [48]. The dentist was well trained and performed at least 80 mercury amalgam removal annually.

### 2.6. Statistical Analysis

All statistical tests, performed with the use of SPSS software, version 19, were two sided, and a *P* value of less than 0.05 was considered to indicate statistical significance. The statistical tests used were chosen after confirmation of the distribution of normality of the sample with the Kolmogorov Smirnov test. Comparison of continuous variables between the two groups was conducted with the use of the Mann-Whitney *U* test for variables with a non-normal distribution. Chi-square tests were used for the comparison of categorical variables and the Fisher's exact test was used where appropriate. The association between two casual variables has been detected with the Correlation Pearson (*r*) test. All data are expressed as means ±SD.

## 3. Results

### 3.1. Patients

**Figure 1:**
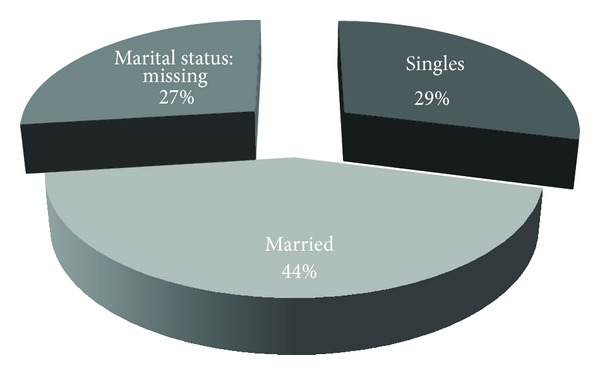
Marital status of the cohort of MCS patients.

The total of 41 patients with MCS were of white race. Patients were of Caucasian adult origin and were identified as Italy-born persons. Most patients were from Northern Italy (80.5 percent), Central Italy (12.2 percent), and Southern Italy (7.3 percent) (Table 2). The mean age of patients with MCS was 44.8 ± 11.2 years; 95.1 percent (39 patients) were female and 2 were men (Table 3). Female mean age was 45 ± 11.3 years and men mean age was 36 years. Details of marital status are shown in Figure 1. The acquisition of MCS was largely associated with female sex; female to male ratio was 19.5 : 1.

### 3.2. Prevalence of Allergy to Metals among Patients with MCS

In the cohort of patients screened with both patch testing and the lymphocyte transformation test (LTT), the cumulative prevalence of allergic sensitization to metals in 26 patients (26 of 41, 63.4 percent) was 92.3 percent. In order to compare the frequency of allergy to metals using the two methods, patch testing for dental series was assessed in 21 of our series of 41 patients (51.2 percent). Positive allergic patch test reactions to metal allergens were noted in 17 of 21 participants (80.9 percent). The prevalence of positive reactions to metals by the lymphocyte transformation test (LTT) was 94.4 percent (17 of 18). 5 patients (5 of 18, 27.7 percent) refused patch testing for contact allergy procedures to establish possible sensitization to dental materials because of contact with metal allergens and the subsequent risk of flare-up of MCS symptoms, therefore, they choose to use another laboratory test: an *in vitro* testing, the lymphocyte transformation test (LTT). In 13 patients (13 of 18, 72.2 percent) sensitization to metals was reported with the use of both methods—skin patch testing and the LTT. When assessed according to mercury-compound allergens by patch testing with a dental screening series (in aggregate; metallic mercury, ammoniated mercury, thimerosal, phenyl mercury, and mercury dental amalgam, all of which in petrolatum) and/or the lymphocyte transformation test (LTT), allergy to mercury was diagnosed in 13 patients of 26 (50 percent). We also found that 22 patients of 26 (84.61 percent) had allergy to other metallic components of dental amalgam, which is the metal-matrix alloy of dental amalgam. By excluding mercury compound allergens, the most common metal immune hypersensitivity reactions associated with amalgam metal-matrix alloy were—listed in decreasing order of frequency—nickel, cadmium, palladium, gold, chromium, and silver.

### 3.3. Mercury Analyses

Of 19 patients who could be evaluated, in 19 of 41 (46.3 percent) the mean mercury whole blood levels was 7.6 ± 13.6 micrograms per liter (range from 0.5 to 59.4 micrograms per liter); normal range from 0 to 2. Of 19 patients considered, in 19 of 41 (46.3 percent), the mean of urine mercury levels was 1.9 ± 2.5 micrograms per liter (range from 1 to 10 micrograms per liter); normal range from 0 to 2. Of 17 patients evaluated, in 17 of 41 (41.5 percent), the mean of total mercury accumulated in scalp hair was 2.2 ± 2.5 micrograms per gram (range from 0.06 to 8.45 micrograms per gram); normal range from 0 to 2. Of 20 patients evaluated, 20 of 41 (48.8 percent), the mean of salivary mercury levels in chewing gum-stimulated whole saliva was 38.1 ± 52.1 micrograms per liter (range from 0.1 to 168 micrograms per liter); upper limit value <2.7 (Table 3). In a subgroup analysis of 24 patients, 22 patients with mercury dental amalgam fillings (91.7 percent), levels of total mercury in human biological matrices (in aggregate; whole blood, urine, scalp hair, and saliva) correlate with the total number of mercury dental amalgam tooth fillings, as compared with 4 patients (50 percent) without mercury dental amalgams (odds ratio, 11; 95 percent confidence interval, from 1.5 to 81.6; *P* = 0.023) (Table 4). In a subgroup of 27 (27 of 41, 65.8 percent) patients with MCS who carry dental amalgam fillings, we also observed a strong correlation between the number of mercury-containing dental amalgam fillings and the concentrations of total mercury in urine (*r* = 0.71, *P* = 0.002). No other variables were associated with a statistically significant increase in level of total mercury in biological matrices (including fish consumption).

### 3.4. Mercury Amalgam Tattoos and MCS

The prevalence of amalgam tattoo of human oral mucosa at the time of first visit was 17 percent (7 of 41), which was more than twice as high as the prevalence among a sample in the Swedish population in whom the prevalence of amalgam tattoo is remarkably high [49]. Intraoral mercury amalgam tattoos are known to have the highest levels of total mercury (inorganic mercury along with organic mercury) compared to any other tissues and organs in humans [50]. Previous estimates of the prevalence of amalgam tattoo ranging from 0.4 to 8.2 percent in the general population [49]. Amalgam tattoo should be surgically removed in case of allergy to mercury.

### 3.5. Mercury Dental Amalgam as a Risk Factor

41 patients with MCS were evaluated for appropriate treatment of adverse events to dental materials, particularly to exposure to dental metal alloys (i.e., mercury-containing dental amalgam fillings). 27 of 41 patients (65.9 percent) had mercury dental amalgam fillings and 14 of 41 (34.1 percent) did not have mercury amalgam tooth fillings. The average number of mercury amalgam restorations of persons in the cohort who carry dental amalgam was 3.8 ± 2.7, ranging from 1 to 10. In 18 of 27 (66.6 percent) patients with mercury amalgam, the mean duration of exposure to mercury amalgam tooth fillings was 25.1 ± 9.5 years before the onset of definite clinical manifestations of micromercurialism after long-term exposure to mercury dental amalgam.

### 3.6. Adverse Events after Mercury Amalgam Removal

A total of 11 patients (11 of 33, 33.3 percent) of the cohort reported having had at least one major adverse outcome related to dental amalgam removal without safe procedures (Table 5). All 11 patients underwent dental amalgam removal at the various dental centers. Clinical manifestations and adverse outcomes that were considered to be related to toxic effects of acute overexposure to mercury vapors during amalgam-removal treatment were as follows: dysgeusia (metallic taste), constriction of the visual fields (tunnel vision), trigeminal neuralgia, atypical facial pain, burning mouth disorder (BMS), cervical lymphadenopathy, axillary lymph nodes enlargement, bronchial hyperresponsiveness and asthma attacks, skin rashes (salmon-colored and/or pink'rash), headache, lightheadedness, weight loss, vertigo, muscle pain/weakness, fatigue, fever of unknown origin (FUO-body temperature ≥37.5°C). The adverse events reported in each patient are listed in Tables 5 and 6. In the Table 6, these adverse events were associated with long-term exposure to mercury amalgam and/or due to high levels of mercury vaporization emitted during amalgam removal by standard drill-out method, which is no longer recommended [48].

### 3.7. Treatment and Mercury Amalgam Replacement

Elemental and inorganic mercury's biological half-life is rather protracted and ranged from 30 to 90 days, averaging 60 days [51]. Most patients who develop MCS following exposure to mercury amalgams show improvement within one year (mean time to resolution = 6 months), after the removal of the remaining mercury dental amalgams. Usually, best results appear to be achieved one year after the last dental amalgam replacement.

### 3.8. Outcomes

16 of 41 (39 percent) patients with MCS were treated by our dental team, in accordance with the *en bloc technique*, in which we do not touch the mercury amalgam filling with tungsten burr [48]. The level of whole blood total mercury, the level of urinary total mercury, and the concentrations of total mercury in saliva all decreased significantly—fell below measurable levels—following mercury amalgam-replacement within 6 to 12 months in 6 patients. After a mean follow up of 41.3 months (ranged from 13 to 130 months), at the end of total mercury amalgam removal, 10 of 16 (62.5 percent) patients reported that their symptoms had improved markedly (>50 percent), according to the patient's subjective assessment. In 37.5 percent (6 of 16) the condition of these patients improved only moderately (<50 percent) after receiving dental amalgam replacement (Table 7). The patients with allergy to metals had the better prognosis and control of symptoms. No side effects were reported with a safe and effective dental amalgam removal.

### 3.9. Body-Mass Index and MCS

The body-mass index—the weight (kg) divided by the square of the height (m)—did not differ significantly in MCS patients. The overall average body mass index (BMI) of 38 female patients and 2 male patients with MCS was 21.3 ± 3.28 (range min–max: 17–30).

### 3.10. Hormonal Risk Factors and MCS

A subgroup of 13 subjects (13 of 41, 31.7 percent) showed hormone disorders, and—of the patients who could be evaluated—an elevated serum prolactin level was detected in 4 of 13 patients (30.8 percent), which means that the endocrine system was found to be deregulated. This corroborates other research suggesting that there was a positive association between the increase in circulating serum prolactin levels and exposure to mercury. 2 of 4 of these patients had allergy to mercury (metallic mercury and mercury dental amalgam, resp.), and they had elevated levels of mercury in saliva samples (17.6 and 49.6 micrograms per liter, resp.). 5 of 13 patients (38.5 percent) had hypothyroidism. 3 of 13 patients (23.1 percent) had hyperthyroidism. Adrenal gland disorders were seen only in one female patient.

### 3.11. MCS, Fibromyalgia, Chronic Fatigue Syndrome, and EHS

Chronic fatigue syndrome (CFS) as well as fibromyalgia were the most common coexisting conditions (fibromyalgia: 11 of 41, 26.8 percent; CFS: 11 of 41, 26.8 percent, resp.). Of 27 patients evaluated, in 14 of 27 (51.9 percent), electromagnetic hypersensitivity symptoms (EHS) were self-reported.

### 3.12. Demyelinating Disorders and MCS

Two patients (2 of 41, 4.9 percent) received a diagnosis of demyelinating disorders within a few months either before or after the diagnosis of MCS.

### 3.13. Dietary Variables and MCS

In 27 patients of 41 (66 percent) the mean consumption level of fish and/or seafood was 1.7 (8.5 oz) fish serving meal per week. It has been reported that foods and beverages (alcoholic) may alter the level of exposure to elemental mercury (Hg^0^) emitted from amalgams in humans. Of 32 patients evaluated, in 11 of 32 (34.4 percent) the level of alcohol consumption per week was 2.43 ± 1.9. Of 32 patients evaluated, 20 of 32 (62.5 percent), the average of coffee consumption per week was 14.7 ± 7.7. Of 32 patients evaluated, 10 of 32 (31.2 percent), the mean number of tea intake weekly was 3.1 ± 2.5. Of 27 patients evaluated, 11 of 27 (40.7 percent) patients reported that consumption per week of chocolate was 1.1 ± 1. The role of dietary factors (i.e., alcohol, coffee, and tea) in the development of MCS remains to be elucidated.

### 3.14. Smoking Status

Of the 35 subjects who were evaluated for smoking, 4 of 35 (11.4 percent) were current tobacco smokers, 9 were former smokers (25.7 percent), and 22 were nonsmokers (62.9 percent).

### 3.15. Other Laboratory Features

It has been suggested that mercury is able to induce a hematologic immunotoxicity. To address this, we estimated total white blood cells (WBCs) count as a subclinical index of mercury immunotoxicity. Of 19 patients who could be evaluated, 6 of 19 (31.6 percent), the average of white blood cells (WBCs) was 5.9 ± 1.5 10^8^ per liter. There was no evidence of the reduction in leukocyte count. We also tested plasma homocysteine concentrations as a subclinical index of mercury vasculotoxicity. Of 15 patients evaluated, 7 of 15 patients (46.7 percent), the average of serum level of homocysteine was borderline low at 13.8 ± 2.7 *μ*mol per liter; normal level, <14. The immunoglobulin E (IgE) level was mildly increased in 16 patients assessed, 6 of 16 (37.5 percent), the average of IgE was 350.1 ± 181.9 UI per milliliter (normal range 0–100 UI per milliliter). These results are consistent with previous studies that showed a role of mercury in immune activation, enhancing B-cell IgE polyclonal production. Biochemical changes, including changes in the serum (liver) alanine aminotransferase (ALT) levels and aspartate aminotransferase (AST) levels (the latter is more reliable indicator for mercury-induced liver injury), were also not significantly different from normal range in our cohort (data not shown). No significant changes in lead blood concentrations (possible plumbism) were found in 10 (10 of 41, 24.3 percent) patients, and no interaction between mercury and lead blood levels was seen in these patients (data not shown).

### 3.16. Rural-Urban Differences and the Prevalence of MCS

**Figure 2:**
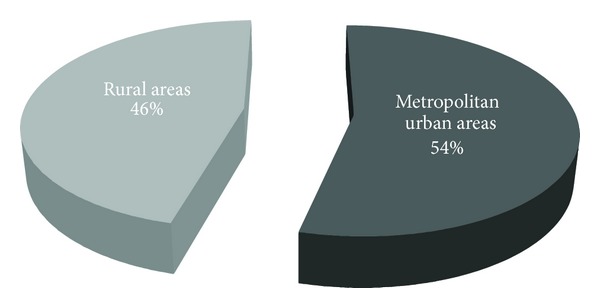
MCS in urban and rural areas.

22 of 41 (53.6 percent) of MCS patients were located in large metropolitan urban areas, where exposure to fine-particulate air pollution has been associated with increased morbidity. 19 of 41 (46.3 percent) were patients who live in rural areas (Figure 2). There was no a statistical difference in the number of patients living in urban compared with rural areas. In two urban Milan (Italy) sites, we measured real-time ambient air mercury vapor concentrations (outdoor Milan atmospheric Hg^0^) and was approximately 50 nanograms per cubic meter, and it was detected by cold vapor atomic fluorescence spectroscopy (CVAFS). Outdoor air levels of mercury in urban area were considerably lower than those released from dental amalgam into the oral cavity in a case series of our patients with mercury amalgam fillings (i.e., 50 nanograms per cubic meter *versus* 15–30 micrograms per cubic meter, during mastication).

### 3.17. Exposure to Pesticides

Some studies have previously highlighted a link between pesticides exposures and the development of MCS [20, 22, 24, 52–55]. We were surprised to see that some of our patients applied in-home and/or outdoor pesticides at their residence, even after the diagnosis of MCS.

### 3.18. Exposure to Pets

Of 28 patients interviewed about lifetime indoor pet exposure, 10 of 28 (35.7 percent) reported exposure to pets, mainly cats (90 percent).

### 3.19. Exposure to Solvents and MCS

Thirty-nine percent (16 of 41, 39 percent) of the cohort reported having had adverse events to organic solvents. In some instances, we witnessed episodes of clinical important aversive response to organic solvents involving respiratory system (bronchial hyperreactivity and asthma attacks) and central nervous system (vasovagal response: fainting and/or near-fainting, dizziness, vertigo, low blood pressure, and tremor) triggered by acute (short-term) inhalation exposures to organic solvents. Of the 28 patients who could be evaluated, (28 of 41, 68 percent) chemical odor intolerance (i.e., hyperosmia, cacosmia, and dysosmia) was self-reported in 24 of 28 (85.7 percent). In some patients the perception of chemical odor (i.e., perfumes, fragrances, hairsprays, cigarette smoke, and diesel fumes) persists some days after exposure.

### 3.20. Dietary Supplements and Antioxidant Supplements

Professionals usually recommended supplements as a supportive therapy to reduce mercury burden. In theory, these supplements would bind and detoxify mercury that can be deposited in parenchymal pattern, reestablishing and maintaining the hepatic glutathione stores (i.e., *N*-acetylcysteine (NAC), selenium, and reduced glutathione, GSH). By contrast, there is no documented evidence either in animal model or in outcome studies that support therapy with supplements is able to remove mercury from human tissues [56]. Our observations raise the possibility that treatment with vitamins, minerals, and antioxidants did not ameliorate the symptoms in most patients with MCS. In particular, we suggest that important clinical adverse events associated with oral supplements should be carefully evaluated when these nonspecific supportive therapies are prescribed to persons who have a clear history of mercury amalgam exposure, for example, ascorbic acid and thiol (sulfur derivatives) agents. Vitamin C (ascorbic acid) was not able to mobilize and remove mercury from tissues in both human and animal studies [56, 57]. Rather, some antioxidants worsen the retention kinetics of mercury in patients exposed to dental amalgam. 5 of 41 (12.2 percent) patients received secondary supportive therapy: 3 patients received selenium, 1 patient received alpha lipoic acid (ALA), and 1 received *N*-acetylcysteine (NAC). Adverse health effects occurred in 3 of 5 (60 percent). In two (2) of them, supplementation with selenium has been implicated in an elevation of the levels of serum antinuclear antibodies (ANAs) and one patient developed severe major aphthous stomatitis, whereas panic attacks were associated with the oral administration of alpha lipoic acid (ALA). 7 of 10 patients who have received intravenous (iv) administration of reduced glutathione (GSH) by continuous infusion, 5 of 7 (70 percent) patients had various adverse events while receiving GSH, including urticaria, asthma attacks, worsening of MCS symptoms, and cheilitis.

### 3.21. Chelation Therapy and MCS

Chelating agent-related toxic effect was reported to us by one patient who underwent a tentative treatment of mercury-chelation therapy. This patient, one of 41 (2.4 percent) received calcium EDTA (ethylenediaminetetraacetic acid—EDTA) by intravenous (iv) continuous infusion [57]. This approach with chelating agents for “detoxification” of mercury was suggested by other physicians, and in our view, it should be used with great caution. Chelation and mobilizing agents are usually contraindicated in patients with mercury-containing dental amalgam fillings [58, 59].

### 3.22. Interactions between Mercury and Other Metals

#### 3.22.1. Mercury and Chromium

In our accumulated clinical experience, chromium allergy and/or chromium exposure appear to confer a considerable susceptibility to mercury exposure. The relation between such biochemical interaction in humans is currently unclear. In our case series of 41, in 4 of 26 patients the point prevalence of allergy to chromium was 15.4 percent.

#### 3.22.2. Mercury and Titanium

We also observed a potential interaction between mercury amalgam restorations and endosseous dental titanium implants among 4 patients in our group with MCS (9.8 percent, 4 of 41). In these 4 patients, we have noted important neurological adverse events (i.e., persistent idiopathic facial pain).

#### 3.22.3. Mercury and Gold

Both metallic mercury (Hg^0^) and gold may elicit autoimmunity in humans and experimental animals. Some early studies suggest that after the removal of mercury dental amalgam serum antinuclear antibodies (ANAs) frequently reverted to negative [60].

## 4. Discussion

A surprisingly very high prevalence of allergic sensitization to metals (92.3 percent) was detected among 26 of 41 patients with MCS in our cohort. Consistent with our findings, other studies have reported a high prevalence of allergy among persons with a diagnosis of MCS [61]. In this study, patients with MCS had significantly higher prevalence of allergy and/or immune sensitization to metals with respect to the general population and were more likely to have both fibromyalgia (FM) and chronic fatigue syndrome (CFS) [62].

Combined skin patch testing and lymphocyte transformation test (LTT) were sufficiently sensitive and specific to provide important clinical guidance.

Importantly, mercury-containing dental amalgam filling was associated with increased odds of elevation of mercury in biological indicator media (blood, urine, saliva, and scalp hair). An unexpected finding of our study is the very high salivary mercury levels (mean 38.1 ± 52.1 micrograms per liter, threshold limit values <2.7) in MCS patients who have mercury-containing dental amalgam. It appears that saliva mercury levels were significantly higher in individuals affected by MCS, as compared with other cohorts. Mercury amalgam tattoo—the clinical hallmark of dental amalgam—was more prevalent among patients with MCS than the population-based prevalence (17 *versus* 8 percent) [49]. The potential toxicity of mercury-containing dental amalgam has been underestimated for a long time in patients with underlying MCS. Nearly 33 percent of the patients were given a diagnosis of MCS after dental amalgam removal treatment, thus, there is a clear evidence that unsafe and inaccurate removal of mercury amalgam is a major risk factor for MCS. According to our experience and on the basis of data from the literature [63], many patients reported symptomatic improvement of MCS after complete removal of mercury-containing dental amalgam as well as other dental alloy restorations (especially palladium- and gold-based alloys) [63].

### 4.1. Mercury Toxicity

Mercury has no known biological role in normal human metabolism. In all of its chemical forms, mercury is able to increase the production of reactive oxygen species (ROS) and the subsequent oxidative stress, potentially causing DNA damage [64]. Mechanisms of mercury toxicity also include inactivation of enzymes (mainly sulfhydryl groups—SH), disruption of membranes, and altered neurotransmitters [56]. A well-known toxic endpoint of mercury is the immune system, in fact, immunotoxic effects on cytokines production (elevated serum interleukin-2 receptor) and autoimmune disorders have been described in the literature [56, 60]. Mercury amalgam is a direct toxicant but it is also a health hazard because of its conversion (biomethylation), owing to oral bacteria biotransformation from inorganic mercury to organic mercury [65]. Experimentally, three chemical species of mercury are present in saliva specimens in individuals carrying mercury-containing amalgam fillings: metallic mercury (Hg^0^), inorganic mercury (mainly inorganic divalent mercury Hg^2+^), and organomercury compounds (as mono methyl mercury—CH_3_Hg^+^—and ethyl mercury—CH_3_CH_2_Hg^+^) [65–67]. Consequently, adverse events to inorganic and/or organic mercury content in saliva may involve both immune and non immune mechanisms.

### 4.2. Caveats about Mercury Biomonitoring

Whole blood and urine mercury levels are believed to be a reliable marker for recent exposure to inorganic and elemental mercury (Hg^0^). Therefore, monitoring blood and urine is valuable for identifying patients with acute exposure to mercury. With ongoing exposures, however, tissue levels of mercury in humans are increased due to accumulation [68], especially in brain (pituitary gland and cerebral cortex), central nervous system, thyroid, and kidneys, as established in previous studies from postmortem examinations [59, 68, 69]. This may elucidate why monitoring blood concentrations of total mercury is of questionable clinical relevance as indicator of tissues body burden of mercury released from dental amalgam in humans. Consistently, preclinical studies have suggested direct evidence that low circulating mercury levels could reflect mercury disposition and redistribution to target organs, at least in adult sheep model [68]. Hence, concentrations of mercury in blood and urine may underestimate retention toxicity of mercury in the tissues and organs. In other words, there is the possibility that measurements of mercury in blood and urine do not fully reflect the actual mercury amalgam burden in humans [59].

### 4.3. Clinical Toxicology of Mercury

Patients with clinical signs and symptoms of unrecognized chronic mercury exposure from dental amalgam fillings—which is frequently overlooked on physical examination—are likely to have a misdiagnosis of postviral syndrome, endocrine disorders, or psychiatric dysfunction [4]. Therefore, due to a lack of specificity, particularly early signs of mercury toxicity, a delay in diagnosis of “micromercurialism” (also known as the “asthenic-vegetative syndrome”) is common, as observed within our cohort. None of the 41 subjects in our present study received a diagnosis of celiac disease (or non-celiac gluten sensitivity) whose condition is associated with increased levels of mercury in both blood and urine [70]. A transient, acute overexposure to mercury vapors (Hg^0^) released from mercury amalgam during drilling cannot rule out the likelihood of long-term health risks [48]. Of note, signs and symptoms caused by chronic exposure to various forms of mercury generated from amalgam are characterized by a very long-lasting latency period of more than 5 or 7 years, as previously investigated [71]. Excluding patients in whom the initiation of MCS symptoms was related to mercury amalgam removal (11 of 33, 33.3 percent), the median times of exposure to mercury amalgam restorations were approximately 20–25 years before the onset of adverse health outcome [72]. With regard to the chronic fatigue syndrome/myalgic encephalomyelitis (CFS/ME) and fibromyalgia conditions (FM), our results are similar to those reported previously, in which several small studies have documented an association between mercury amalgam exposure and CFS/FM [50, 73–76]. In most reported cases of CFS in patients in our series, the features of CFS and the “*asthenic-vegetative syndrome*” caused by inhalation of mercury vapor overlap significantly.

### 4.4. Electromagnetic Hypersensitivity: EHS

The causes of electromagnetic hypersensitivity are not clear and are probably multifactorial, however, we speculate that tissues and organs of the body respond to cellular retention and injury of mercury with inflammation (proinflammatory microenvironment). Exposure to electromagnetic field might increase mercury mobilization of metal ions from tissues, which in turn increases inflammation and oxidative damage to cellular level [77–81].

### 4.5. Mercury in Fish

Additional pathway of exposure to mercury in humans derived from fish consumption, mainly as organic mercury (CH_3_Hg^+^). But although we recognize the potential benefits of eating fish, we are concerned about the possible overlapping toxic effects of elemental mercury (Hg^0^) emitted from dental amalgams and organic mercury exposure (CH_3_Hg^+^) from fish intake. Patients who already have mercury overload from amalgam fillings may be the least able to tolerate additional mercury burden from dietary fish intake and should not be further exposed to organic mercury from fish consumption during amalgam-treatment replacement [82].

### 4.6. Limitations of the Study

There are notable limitations to our study. We did not perform mercury assessments in all the patients and the central limitation is the quantity of missing data. Further, being a retrospective study and as the sample size is fairly small (forty-one case subjects), it should be interpreted with caution.

### 4.7. Interpretation

Studies have documented the increased risks of adverse events associated with exposure to chemical substances among patients with MCS [16, 18–20, 83, 84]. A substantial proportion of our MCS patients (81.2 percent, 26 of 32) had an elevation in the total mercury levels at the time of the first study visit, and they correlated significantly with the total number of mercury-containing dental amalgam fillings. As far as we are aware, this is the first observation of quantitative assessment of the association between increased elevations of mercury in biological indicator media and risk of MCS. It is therefore reasonable to assume that exposure to mercury might contribute to the observed symptoms among patients affected by MCS [53, 54, 85] and that measurements of mercury levels may be clinically useful. It is also not inconceivable that previous exposure to organic solvents (and VOCs) may determine susceptibility to metals in humans, but the mechanisms are not clear. Further studies are needed to document that levels of mercury are increased among individuals with MCS and are able to trigger the disease process.

## 5. Conclusions

This study indicates that there is an increased prevalence of allergy to metals among patients with MCS, whereas a higher level of mercury in biological matrices is associated with the presence of mercury-containing dental amalgam fillings.

## Figures and Tables

**Table 1:** Distribution of patients as stratified according to study cohort and the total number [mean (±SD)] of mercury-containing dental amalgam fillings.

Sex - no. (%)		
Female	39	95.1%
Male	2	4.9%
Patients with dental amalgam fillings	27	65.9%
Mean age	42.4 ± 10.5	
No. mercury amalgam fillings		
Mean	3.8 ± 2.7	
Patients without dental amalgam fillings	14	34.1%
Mean age	49.3 ± 11.7	

**Table 2:** Distribution of 41 cases of multiple chemical sensitivity in the Italy cohort. The MCS cohort contained 39 (95.1 percent) women and 2 (4.9 percent) men.

Regions of Italy	Patients no.	% of total
Lombardy	23	56.10
Emilia-Romagna	4	9.75
Lazio	5	12.19
Trentino-Alto Adige/Südtirol	3	7.32
Veneto	3	7.32
Apulia	1	2.44
Campania	1	2.44
Calabria	1	2.44

**Table 3:** Mean [±SD] total mercury concentrations in biological matrices (whole blood, urine, saliva, and scalp hair).

Characteristics	Total cohort with MCS patients		Concentrations of total mercury in biological matrices micrograms/liter	Reference range micrograms/liter
	41			
Sex - no. (%)				
Female	39	95.1%		
Male	2	4.9%		
Age at diagnosis of MCS				
Mean	44.8 ± 11.2			
Range (min–max)	25–65			
Total cases of Hg in blood	19			
Mean			7.6 ± 13.6	≤2.0
Total cases of Hg in urine	19			
Mean			1.9 ± 2.5	≤2.0
Total cases of Hg in saliva^§^	20			
Mean			38.1 ± 52.1	≤2.7
Total cases of Hg hair analysis	17			
Mean			2.2 ± 2.5	≤2.0*

^§^Total mercury was measured in gum-stimulated saliva samples.

*Total mercury in scalp hair was expressed in micrograms per gram.

**Table 4:** Increased levels of mercury in biological matrices in patients with MCS who have mercury dental amalgams.

	Patients with mercury amalgam fillings	Patients without mercury amalgam fillings	Total patients	*P* value	Odds ratio
Number	24	8	32	0.023	11
Yes	22 (91.7%)	4 (50.0%)	26 (81.3%)		
No	2 (8.3%)	4 (50.0%)	6 (18.7%)		

In a subgroup analysis of 24 patients, 22 patients with dental amalgam (91.7 percent), levels of total mercury in biological matrices (in aggregate; whole blood, urine, scalp hair, and saliva) correlate with the number of mercury dental amalgam tooth fillings, as compared with 4 patients (50 percent) without dental amalgams (odds ratio, 11; 95 percent confidence interval: from 1.5 to 81.6; *P* = 0.023).

**Table 5:** Adverse health effects occurring in 33.3 percent of patients (11 of 33) who underwent mercury dental amalgam removal without adopting safety measures.

Patient no.	Sex	Age at diagnosis	No. of total mercury amalgam fillings	Reported adverse events	Allergy to metals*	Blood mercury levels *μ*g/L	Urine mercury levels *μ*g/L	Scalp hair mercury levels *μ*g/g	Saliva mercury levels *μ*g/L
1	F	52	PR^‡^	Fever of unknown origin	Thimerosal	0.5	0.5	0.06	NA**
2	F	31	2	Asthma attacks	Nickel	2.7	0.7	NA	0.7
3	F	27	2	Asthma attacks	Thimerosal nickel	59.4	4	8.2	1.3
4	M	36	PR	Tunnel vision, trigeminal neuralgia, metallic taste	Inorganic mercury	NA	NA	0.2	0.2
5	F	39	PR	Hyperosmia, asthma attacks	NA	NA	NA	NA	NA
6	F	29	5	Fever of unknown origin	NA	1.1	0.3	NA	2.4
7	F	61	2	Atypical facial pain	NA	NA	NA	2.8	2
8	F	31	PR	Fatigue, muscle pain	Nickel	NA	NA	NA	NA
9	F	58	1	Facial paresthesia, metallic taste, ocular inflammation	NA	7.6	0.5	4.03	0.7
10	F	47	2	Vertigo, asthma attacks, pricking pain in arms	NA	2.7	0.5	1.3	NA
11	M	36	2	Fatigue, muscle pain/weakness	Inorganic mercury	3.5	0.5	NA	9.2

*Allergy to mercury: patients were tested with skin patch testing and/or lymphocyte transformation test (LTT).

^‡^PR: previously removed, number of mercury amalgam undefined.

**NA: not analyzed.

**Table 6:** New and classical systemic signs and symptoms associated with mercury exposure among MCS patients cohort.

Signs	
(i) Angioedema	N
(ii) Cervical and axillary lymph nodes swollen	N
(iii) Dermographism	C
(iv) Enlargement of thyroid	C
(v) Eyelid myokymia (eyelid tremors)	N
(vi) Gastrointestinal malabsorption	N
(vii) Gingivitis - Stomatitis	C
(viii) Lichenoid contact stomatitis	N
(ix) Low-grade fever (fever of unknown origin—FUO)	N
(x) Muscle atrophy	N
(xi) Muscle fasciculations	C
(xii) Non-allergic rhinitis/vasomotor rhinitis-like	N
(xiii) Peripheral neuropathy	C
(xiv) Salmon-colored and/or pink' rash	C
(xv) Sialorrhea (hypersalivation)	C
(xvi) Spasms	C
(xvii) Systemic contact dermatitis	N
(xviii) Tremors (upper limb, hands, fingers, face, eyelids, and lips)	C
(xix) Urticaria	N
(xx) White matter hyperintensity (by brain MRI)	N
(xxi) Xerostomia (dry mouth)	C

Symptoms	

(i) Abdominal cramps	N
(ii) Anorexia	C
(iii) Atypical facial pain (persistent idiopathic facial pain)	N
(iv) Burning mouth syndrome (BMS)	N
(v) Burning pain (neuropathic)	C
(vi) Chemical odor intolerance	N
(vii) Chest pain (anterior or posterior, on the left side)	N
(viii) Confusion	C
(ix) Depression	C
(x) Dysesthesia	N
(xi) Fatigue	C
(xii) Flu-like symptoms	N
(xiii) Headache	C
(xiv) Insomnia	N
(xv) Intestinal movement disorders	N
(xvi) Intolerance to odors	N
(xvii) Itching (neuropathic)	N
(xviii) Muscle weakness	C
(xix) Nausea	C
(xx) Noise sensitivity	N
(xxi) Paresthesia	C
(xxii) Photophobia	N
(xxiii) Recurrent infections	N
(xxiv) Short-term memory disturbances	C
(xxv) Tachycardia	C
(xxvi) Thermal regulation disorders (low cold tolerance)	N
(xxvii) Trigeminal neuralgia	N
(xxviii) Vertigo	C

The table lists signs and symptoms triggered by mercury amalgam exposure and also noting those defined as “new” and “classical” signs and symptoms related to exposure to mercury amalgam in a cohort of patients with MCS. (N) and (C) denote “new” and “classical” signs and symptoms.

**Table 7:** Recommended threshold levels in matrices for biological monitoring of total mercury in humans after complete mercury dental amalgam removal. Very low level of mercury in bioindicators are able to reverse clinical manifestations as well as abnormal laboratory values associated with mercury amalgam exposure.

Mercury levels	Threshold limit values	Unit of measurements
Total Hg in scalp hair	≤0.5	micrograms/g
Total Hg in whole blood	<1.5	micrograms/L
Total Hg in serum	<1.5	micrograms/L
Total Hg in plasma	<1.5	micrograms/L
Total Hg in urine	≤1.0	micrograms/L
Total Hg in saliva	≤0.5	micrograms/L
Total Hg in breast milk	≤0.5	micrograms/L
Total Hg in nails	≤0.5	micrograms/g
Total Hg in intraoral cavity	≤1.5	micrograms/m^3^
